# Female Brown Long‐Eared Bats (*Plecotus auritus*) Delay Roost Emergence at Elevated Natural Light Conditions

**DOI:** 10.1002/ece3.71699

**Published:** 2025-06-29

**Authors:** Mari A. Fjelldal, Jonathan Wright, Thomas M. Lilley, Rune Sørås, Clare Stawski

**Affiliations:** ^1^ Finnish Museum of Natural History University of Helsinki Helsinki Finland; ^2^ Norwegian University of Life Sciences Environmental Sciences and Natural Resource Management Ås Norway; ^3^ Department of Biology Norwegian University of Science and Technology Trondheim Norway; ^4^ Swedish Biodiversity Centre Swedish University of Agricultural Sciences Uppsala Sweden; ^5^ School of Science, Technology and Engineering University of the Sunshine Coast Maroochydore DC Queensland Australia

**Keywords:** emergence time, foraging, high latitude, light‐averse, nocturnal, reproduction

## Abstract

Nocturnal animals inhabiting northern latitudes face prolonged periods of reduced foraging times in summer due to short light nights. The energetic challenges of reduced foraging times are further heightened in reproductive mammals that allocate substantial resources to offspring care with peak energy demands in mid‐summer. However, little is known about responses to variation in natural light conditions at high latitudes in light‐averse species, such as slow‐flying gleaning bats, especially during reproduction. Here, we investigate the impacts of natural light levels and other environmental conditions (i.e., temperature, rain and wind) on individual‐level activity patterns (emergence time, return time, proportion of night utilised) in reproductive and non‐reproductive female brown long‐eared bats, *Plecotus auritus* (*N*_ind_ = 27) in Norway (60.1° N) collected across three summers (2019–2021). We found that bats delayed the start of evening foraging trips on lighter nights, typically emerging from the roost only when light levels decreased below 5 lux, likely because higher light levels are associated with increased predation risk. However, no such effect was found in morning return times to the roost, for which bats showed greater light tolerance. Lactating females took apparently higher risks and left the roost approximately 20 min earlier than non‐reproductive females, presumably because of their greater energetic requirements. They also spent a larger proportion of the night away from the roost compared to pregnant and non‐reproductive individuals, although this proportion was influenced by variation in environmental conditions, such as temperature, rainfall and windspeed. Our results highlight the dynamic nature of responses in light‐averse bats balancing risks of predation against foraging gains during reproduction at northern latitudes. Reduced foraging times during short northern nights may thus represent a hard constraint to range expansion in slow‐flying gleaning bats, even if other environmental conditions improve with climate change.

## Graphical abstract

Here we study responses to natural light conditions in foraging patterns of a light‐averse bat species ( *Plecotus auritus* ) during the energetically expensive reproductive period. Our results highlight that: (1) some bats are more light‐averse than others, which can be related to flight technique and foraging strategy, (2) responses to light depends on reproductive state of the animal and (3) strong light‐aversion in nocturnal species could have implications for future geographical distribution expansions at northern latitudes.

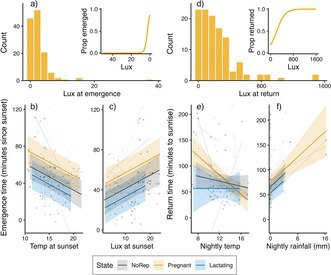

## 1 Introduction

At northern latitudes, seasonality dictates the circannual rhythms of animals (Gwinner 2012). The higher temperatures and longer days during summer increase primary production and provide organisms with sufficient resources and energy to reproduce. However, short summer nights present a conundrum for nocturnal mammals inhabiting sub‐polar regions by limiting their time to be active and forage (Daan and Aschoff 1975). This is emphasised in insectivorous mammals, such as bats, for which the season represents an abundance of food, but only for a limited time daily due to the short and light nights (Rydell 1992; Fjelldal et al. 2023).

Nocturnality in bats has likely evolved as a strategy to exploit vacant ecological niches whilst avoiding diurnal avian predators and resource competitors (Speakman 1991b; Rydell and Speakman 1995; Speakman 1995). Bats are therefore rarely observed flying in daylight, and individuals time their evening emergences from and morning returns to their day‐time roosts depending upon environmental factors, such as current weather conditions. Additionally, the foraging ecology of different bat species also impacts this timing to varying degrees. For example, faster aerial hawking taxa, that are more dependent upon the flight activity of small dipterans at dusk, tend to exit roosts earlier (i.e., under brighter conditions) than slower flying gleaning taxa, which can make better use of nocturnal moths and terrestrial arthropods in their diet (Jones and Rydell 1994). Therefore, aerial hawking species can be considered pre‐adapted to short and light boreal nights, whereas the activity of slower flying gleaning taxa is more strictly governed by prevailing light conditions (Jones and Rydell 1994). For instance, brown long‐eared bats ( *Plecotus auritus* ) emerge from their daytime roosts late in the evening, as compared with other sympatric bat species (Jones and Rydell 1994; Entwistle et al. 1996), as well as avoiding streetlights and illuminated buildings (Rydell et al. 2017; Reusch et al. 2024). This light‐averse behaviour could be due to higher predation risks from potential predators, given that it is a relatively slow‐flying species (Norberg 1976; Jones and Rydell 1994). Light‐averse responses in bats have been described in several studies investigating the effect of artificial light at night (e.g., Voigt et al. 2021; Reusch et al. 2024). There is, however, a lack of information on the impacts of natural daylight on such light‐averse bats in high‐latitude environments, even though the bright light conditions during sub‐arctic summer nights have previously been suggested to limit the northern ranges of many bat species (Parker et al. 1997; Slough and Jung 2008; Fjelldal et al. 2023).

Some species of bats have distributional ranges that extend up towards, and even above, the Arctic Circle (Slough and Jung 2008; Tidenberg et al. 2019; Suominen et al. 2022), where the shortest and brightest nights in mid‐summer coincide with the timing of parturition in high‐latitude bat species (Linton and Macdonald 2018; e.g., Lilley et al. 2025). Pregnant and lactating females therefore face a period of severely restricted foraging opportunities just when they are experiencing peak energy demands (Kurta et al. 1989; Rydell 1993). This challenge is further intensified by summers in the north being associated with unstable weather conditions and nights that can be cold, wet and windy, all of which have been found to delay evening emergences (Suutari et al. 2024), reduce growth in juveniles (Linton and Macdonald 2018; Davy et al. 2022; Fjelldal and van der Kooij 2024) and decrease foraging activity in bats (Wolcott and Vulinec 2012; Fjelldal et al. 2021), likely due to increased flight costs and/or reduced prey availability (Griffin 1971; Tuttle and Stevenson 1982; Norberg 1990; Speakman et al. 2000). To overcome the energetic challenges at northern latitudes, insectivorous bats are capable of employing torpor opportunistically during summer to save energy during daytime or when foraging conditions are poor (Fjelldal et al. 2023). However, because torpor slows fetal development (Racey and Swift 1981; Dzal and Brigham 2013) and reduces milk production (Wilde et al. 1999; Geiser 2021), reproductive females tend to restrict the duration and/or depth of torpor use (Dzal and Brigham 2013). Thus, their only remaining solution is to alter their foraging behavior to accommodate heightened energetic needs.

Previous studies have found that lactating female bats at northern latitudes increase diurnal foraging durations (Rydell 1993; Duvergé et al. 2000; Lilley et al. 2025), likely to meet the high energetic demands of milk production (Kurta et al. 1989). For example, lactating northern bats ( *Eptesicus nilssonii* ) increased their foraging activities through a combination of earlier emergence and later returns to the roost, and were less influenced by nightly temperatures compared to pregnant bats (Lilley et al. 2025). However, *E. nilssonii* is the northernmost breeding bat species in the world and considered a light‐tolerant species (Frafjord 2021). On the other hand light‐averse species, such as *P. auritus* , are likely more restricted by light conditions in high latitude environments, which could be a limiting factor in expanding their distribution range farther north, even if climate change was to improve other limiting environmental factors. Such limitations may negatively impact the species if their southern range contracts due to the changing climate. However, we still lack knowledge on the responses of light‐averse bat species to natural light conditions in high‐latitude environments and on how individual reproductive states influences the dynamics of activity patterns in such bat species during short summer nights.

We aimed to statistically disentangle the different factors incorporated into the activity patterns in a northern population (60° N) of reproductive and non‐reproductive female *P. auritus* , by analysing variation in individual timing of emergence from and return to the day‐time roost, and overall utilisation of the time between sunset and sunrise in response to environmental conditions (lux, temperature, rainfall, windspeed and barometric pressure). Despite the already short time available to forage in mid‐summer at this high‐latitude location, we predict that slow‐flying *P. auritus* will show clear signs of light‐aversion by delaying emergences on brighter evenings and by returning earlier on brighter mornings. However, in accordance with observations from studies on sympatric bat species (e.g., *Rhinolophus ferrumequinum* : Duvergé et al. 2000; *E. nilssonii* : Lilley et al. 2025), we expect lactating females with their increased energetic demands to risk earlier emergence and to stay out longer compared to non‐reproductive females, as well as perhaps to heavier pregnant females with their increased flight costs. Due to the potential energetic mismatches between flight costs and available resources when weather conditions are poor, we further predict that the overall proportion of the night utilised will generally decrease during poor environmental conditions, such as lower temperatures, stronger winds and heavier rainfall. However, we expect lactating females to be less sensitive to changes in environmental conditions given their heightened energetic needs. Our analyses will thus determine how reproductive and non‐reproductive females balance their energy budgets in summer, despite the challenges of a high‐latitude environment. Such findings can shed light on the adaptability of high‐latitude populations of bats and might indicate potential limitations to any future expansion of the distributional range of species further north in response to climate change.

## 2 Methods

### 2.1 Study Species

We investigated individual strategies in summer foraging behaviour across reproductive states in high latitude *P. auritus* females in Norway (60.05° N, 10.87° E). *Plecotus auritus* is a small (6–12 g) gleaning bat in the Vespertilionidae family that feeds mainly on nocturnal moths (Vesterinen et al. 2018) and is a common species throughout Europe, with a geographical northern distributional limit around 63°–65° N (Ancillotto and Russo 2023). The reproductive cycle of *P. auritus* follows that of other temperate zone bat species with a mating period during autumn and winter, after which the spermatozoa are stored in the female reproductive tract throughout the winter hibernation period (Burland et al. 2001). Fertilization happens after emerging from hibernation in spring, and females give birth to a single pup (although twinning can occur) in summer. Mixed sex maternity colonies tend to form during the gestation and lactation period, but the colonies disperse towards the approaching mating period in autumn (Stebbings 1966; Entwistle et al. 2000). Summer roosts of *P. auritus* can be found in tree cavities, bat boxes, and lofty spaces in buildings, such as attics, barns and churches, with colony sizes commonly ranging between 5 and 60 adults (Entwistle et al. 1997, 2000; Ancillotto and Russo 2023). Known predators include tawny owls ( *Strix aluco* ) and barn owls ( *Tyto alba* ) (Glue 1974; Speakman 1991a; Ancillotto and Russo 2023). However, diurnal raptors such as falcons and hawks are also known to hunt small bats opportunistically (Speakman 1991a; Mikula et al. 2013). We observed tawny owls on a few occasions during our fieldwork, although we never observed any predation attacks on bats.

### 2.2 Study Area and Data Collection

Permits to conduct this study were granted by the Norwegian Food Safety Authority (FOTS ID 23284) and the Norwegian Environment Agency (ref. 2018/4899). We collected data during three consecutive summers (2019–2021) from early June to the beginning of August at a study site in Nittedal, Norway (60.05° N, 10.87° E). The study site is situated in a valley which stretches from north to south. We captured *P. auritus* using mist‐nets erected across tree‐corridors and within forest openings, normally from 30 min before sunset to 1–3 h after. The nets were monitored continuously to ensure that no captured bat would hang in the net for prolonged periods of time, as well as to record the exact capture time. After capture, we recorded individual forearm length, body mass and sex. Females were further checked for signs of reproduction, which included pregnancy (by gently palpating their abdomen), lactation, post‐lactation, or we recorded that they showed no apparent reproductive signs. Only one tagged female showed signs of post‐lactation, and for the analyses we therefore recorded this individual as ‘non‐reproductive’.

After the initial processing, we fitted small transmitters (0.4 g, PIP31; Lotek Wireless Inc., Dorset, UK, weighing ~5% of individual body mass; mean: 5.3% ± 0.9% SD) to the captured females (*N* = 28) by trimming a patch of fur from the dorsal region and attaching the tags using a skin adhesive (B‐530 Adhere Adhesive or Sauer‐Hautkleber 50.01; Manfred Sauer GmbH, Lobbach, Germany). We then released the tagged individuals and tracked them to their day roosts using radiotelemetry. At each individual day roost (total *N* = 19; tree roosts = 14, building roosts = 5; however, the building roosts were utilised more frequently and by a larger number of the individuals), we placed data loggers (G. Körtner, Armidale, Australia) in close proximity (< 10 m) to the tagged bats, recording signals from the transmitters every 10 min. When bats flew out of the roosts at night the signals disappeared, thus recording periods of presence and absence. We collected data from a total of 27 females (*N*_ind_ = 15 in 2019; *N*_ind_ = 8 in 2020; *N*_ind_ = 4 in 2021), because data from one female had to be excluded due to problems related to the transmitter signal drifting. Of the 27 females, we recorded 8 as ‘non‐reproductive’, 11 as ‘pregnant’ and 8 as ‘lactating’. The recordings lasted until bats shed their transmitters (recording duration ranging from 1.5 to 19 days; median = 5.5 days).

Throughout each field season, light measurements (lux) were recorded every 10 min by a light logger (Illuminance UV recorder TR‐74Ui; T&D Corporation, Tokyo, Japan) that we placed in an open area close by the field site (from 0.2 to 5 km away from any of the day roosts). Air temperature (°C) outside each roost was recorded every 10 min by small temperature loggers (0.5°C, DS 1921G Thermochron iButtons; Maxim Integrated Products Inc., Sunnyvale, CA, USA) that we hung inside paper cups (to avoid wind chill or direct sunlight effects) from tree branches 1.5–2.0 m above the ground. We obtained environmental data on rain (mm, cumulative last 10 min), wind speed (m/s, mean wind speed last 10 min) and barometric pressure (hPa, hourly) from the weather station in Hakadal (station number SN4460) through the Norwegian Centre for Climate Services webpage (klimaservicesenter.no). The weather station is located in the same valley, approximately 7 km north of the study area. Timing of sunset and sunrise were obtained through the Time and Date webpage (Timeanddate.com).

### 2.3 Timing of Emergence

We defined the timing of emergence as the time of the first missing datapoint from the telemetry tag during the nightly absence period for each female per night. We then calculated the timing of emergence relative to sunset (i.e., higher values indicating later emergences) to use as the response variable in statistical analyses. To investigate the causes of variation in emergence time (minutes since sunset), we constructed a global linear mixed effect model using the *lmer* function from the lmerTest package (Kuznetsova et al. 2017) in the statistical software R (version 4.4.2). Fixed effects included in the global model were individual reproductive state, mean temperature the hour before sunset, mean rainfall the hour before sunset, mean windspeed the hour before sunset, barometric pressure at sunset, lux levels at sunset and night length (hours from sunset to sunrise). Because light levels were only recorded every 10 min, and the light can change drastically during 10 min at dusk, we interpolated light condition values for any sunset that did not fall exactly on the minute of the light level recordings. We tested for interactions between reproductive state and each environmental predictor, except rainfall which was included as an additive effect due to little variation within this variable. Individual ID, date and roost ID were included as random effects. We performed model selection on the global model by applying the *dredge* function from the MuMIn package (Barton 2022). If the highest ranked model had an AICc value < 2 of any of the lower ranked models, we chose the model with the fewest degrees of freedom as our best model, based on parsimony. Multicollinearity between predictors in the final model was evaluated using the *vif* function from the car package (Fox et al. 2012), ensuring that the variance inflation factor of each predictor was < 5, and normal distribution of the model residuals was confirmed. Finally, to disentangle within‐ versus among‐individual effects of explanatory variables in the final model (i.e., after model selection), we applied the mean‐centring methods described in van de Pol and Wright (2009) for distinguishing the effects of within‐individual plasticity versus among‐individual differences.

### 2.4 Timing of Return

We defined the timing of return as the time of the first recorded datapoint after the nightly absence period for each individual per night. We then calculated timing of return relative to sunrise (i.e., higher values indicating earlier returns) to use as the response variable in statistical analyses. The global model explaining variation in the time of return to the roost (minutes until sunrise) included the fixed effects of reproductive state, lux levels at sunrise, mean nightly temperature, mean nightly barometric pressure, total nightly rainfall, mean nightly windspeed and night length (hours from sunset to sunrise), with individual ID, date and roost ID included as random effects. We tested reproductive state in interaction with each environmental variable, except for rainfall which was included as an additive effect. We followed the same procedure for model selection, for evalutating predictor multicollinearity and model residual distribution, and for distinguishing within‐ versus between‐subject effects as described for timing of emergence.

### 2.5 Proportion of Night Utilised

We defined the proportion of night utilised as the total time between an individual's emergence and return to the roost divided by the night length. Signals from the females occasionally appeared briefly during the night in between emergence and return; however, as we could not differentiate between the cause of these erratic signals appearing (females returning to the roost versus foraging in close proximity) we chose to only consider the total time from first emergence to last return as a measure of the total nighttime deemed suitable for activity by the females. The global model for explaining variation in the proportion of the night utilised included the fixed effects of reproductive state, mean nightly temperature, mean nightly barometric pressure, total nightly rainfall, mean nightly wind speed and night length (hours from sunset to sunrise), with individual ID, date and roost ID included as random effects. We tested reproductive state in interaction with each environmental variable, except for rainfall, which was included as an additive effect. We followed the same procedure for model selection, for evaluating predictor multicollinearity and model residual distribution, and for distinguishing within‐ versus between‐subject effects, as described for timing of emergence.

## 3 Results

### 3.1 Timing of Emergence

**FIGURE 1:**
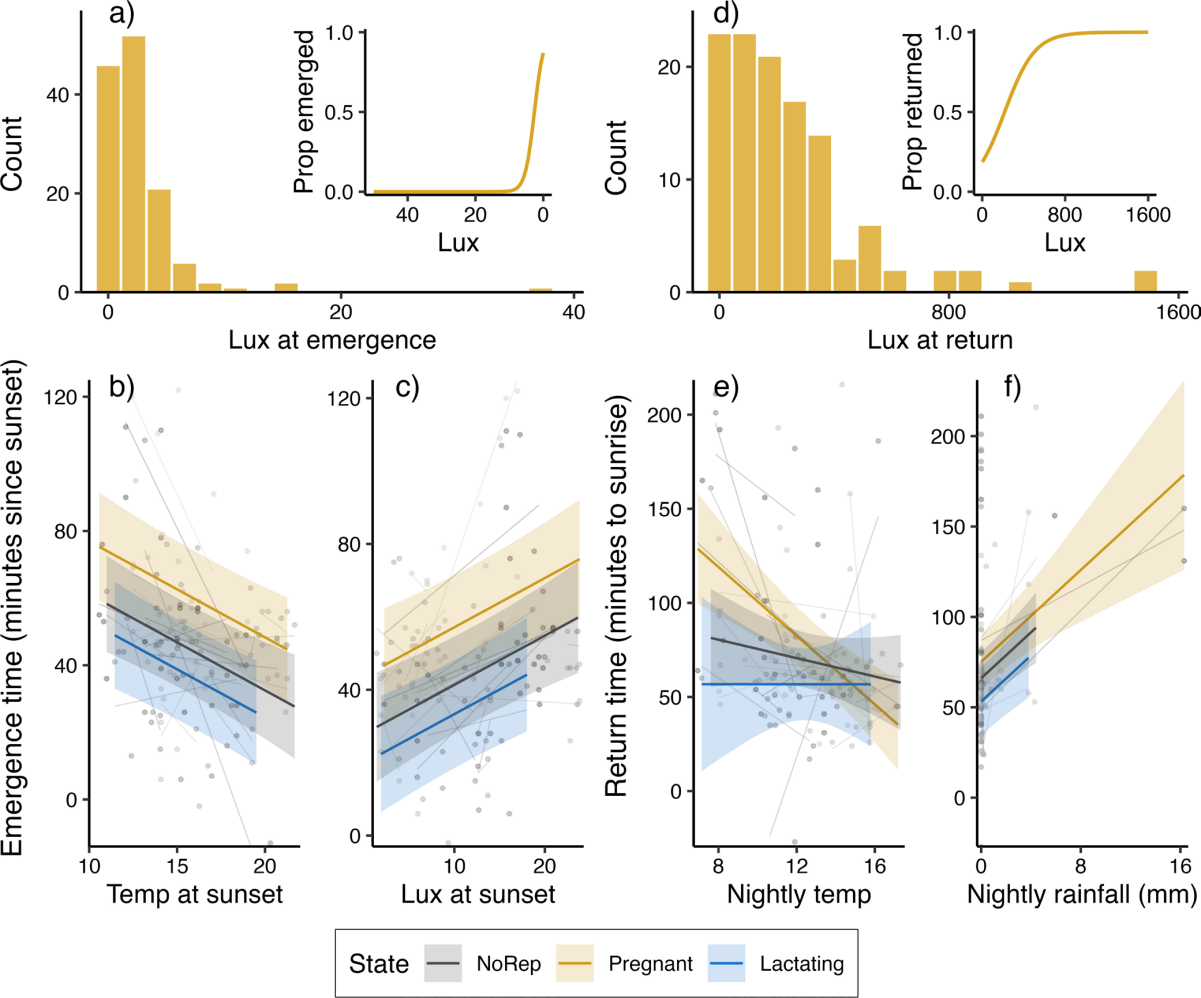
Drivers of the timing of emergence and return in female *Plecotus auritus* . (a) Distribution plot of light levels (lux) measured during emergence from roost. The inset plot shows the proportion of emergences recorded with decreasing light levels in the evening (note the direction of the *x*‐axis). (b) Mean predicted effects and CIs of variation in timing of emergence relative to sunset (higher values = later emergences) explained by temperature at sunset and individual reproductive state. Grey lines and datapoints indicate individual responses (raw‐data not accounting for other effects from the model). (c) Mean predicted effects and CIs of variation in timing of emergence explained by lux at sunset and individual reproductive state. (d) Distribution plot of light levels (lux) measured during returns to the roost. The inset plot shows the proportion of returns with increasing light levels towards morning. (e) Mean predicted effects and CIs of variation in timing of return relative to sunrise (higher values = earlier returns) explained by mean nightly temperature and individual reproductive state. (f) Mean predicted effects and CIs of variation in timing of return explained by total nightly rainfall and individual reproductive state.

We recorded 139 bat emergences from daytime roosts, which involved 27 individual female bats over 75 different nights. Timing of emergence from the roost at night varied from 13 min before sunset to 129 min after (overall mean emergence time: 48 min after sunset ± 23.9 SD). Non‐reproductive females had a mean emergence time (not accounting for environmental effects) of 48 min after sunset (±21.5 SD; *N*_obs_ = 52), while pregnant bats emerged on average 57.8 min after sunset (±25.2 SD; *N*_obs_ = 57) and lactating females 30.1 min after sunset (±13.2 SD; *N*_obs_ = 30). The measured light‐levels at emergence from the roost ranged from 0.07 up to 37.3 lux (median: 1.8 lux), where 90% of all emergences were recorded after light levels decreased below 5 lux (Figure 1a). The best final model (after model selection) explaining variation in emergence time included reproductive state, light level at sunset, mean temperature during the hour before sunset and night length (Table 1a; Figure 1b,c). Lactating females emerged the earliest and pregnant females the latest, while all bats delayed emergences on bright evenings, emerged earlier on warmer evenings, and slightly earlier on short nights. The effects of light levels, temperature conditions and night length on emergence time were all almost completely within‐subject effects of individual plasticity (Table S1).

**TABLE 1:** Model results presenting the final best‐fit model (after model selection) explaining: (a) timing of emergence; (b) timing of the return; and (c) proportion of night spent away from the roost.

Variable	Random effects	Fixed effects	
	Variance (SD)	Estimate (SE)	*p*
(a) Timing of emergence (minutes since sunset)			
Individual ID	39.8 (6.3)		
Date	0.0 (0.0)		
Roost ID	293.5 (17.1)		
Residual	252.8 (15.9)		
Intercept (non‐rep)		−18.0 (46.3)	0.6983
Intercept (pregnant)		15.8 (7.1)	0.0324
Intercept (lactating)		−8.0 (7.3)	0.2766
Temp at sunset		−2.9 (0.7)	< 0.001
Lux at sunset		1.3 (0.3)	< 0.001
Night length		16.4 (7.6)	0.0337
(b) Timing of return (minutes until sunrise)			
Individual ID	110.7 (10.5)		
Date	335.9 (18.3)		
Roost ID	38.2 (6.2)		
Residual	873.5 (29.6)		
Intercept (non‐rep)		96.4 (28.5)	0.0012
Intercept (pregnant)		92.0 (39.2)	0.0215
Intercept (lactating)		−43.2 (56.6)	0.4475
Mean nightly temp		−2.4 (2.2)	0.2683
Total nightly rainfall		6.3 (1.6)	< 0.001
Temp × pregnant		−6.7 (3.0)	0.0281
Temp × lactating		2.4 (4.4)	0.5835
(c) Proportion of night spent away from roost			
Individual ID	0.002 (0.04)		
Date	0.01 (0.12)		
Roost ID	0.0 (0.0)		
Residual	0.006 (0.08)		
Intercept (non‐rep)		0.64 (0.15)	< 0.001
Intercept (pregnant)		−0.51 (0.19)	0.0077
Intercept (lactating)		−0.09 (0.20)	0.6522
Mean nightly temp		0.004 (0.009)	0.6592
Total nightly rainfall		−0.03 (0.006)	< 0.001
Mean nightly wind		−0.01 (0.04)	0.7242
Temp × pregnant		0.03 (0.01)	0.0049
Temp × lactating		0.03 (0.01)	0.0439
Wind × pregnant		−0.03 (0.06)	0.6026
Wind × lactating		−0.19 (0.06)	0.0035

*Note:* Intercepts and their *p* values are given in relation to the first listed intercept value, while interaction effects and their *p* values for pregnant and lactating females signifies whether the effects are different from the effect for non‐reproductive females. See Section 2 for further detail.

### 3.2 Timing of Return

We recorded 123 returns across 72 dates and 26 individuals. Timing of return to the roost at night varied from 216 min before sunrise to 27 min after (overall mean return time: 68.6 min before sunrise ±39.2 SD). Non‐reproductive females had a mean return time of 72.3 min prior to sunrise (±46.0 SD; *N*_obs_ = 49), while pregnant females on average returned 80.1 min before sunrise (±45.5 SD; *N*_obs_ = 51) and lactating females 53.3 min before sunrise (±26.6 SD; *N*_obs_ = 23). Measured light‐levels at the return to the roost ranged from 0.05 up to 1476 lux (median: 177.8 lux), where 90% of all returns were recorded before light levels increased above 600 lux (Figure 1d). The best final model (after model selection) included total nightly rainfall (bats returned earlier on rainy nights), and an interaction effect between reproductive state and mean nightly temperature; while non‐reproductive and lactating individuals were not significantly affected by temperature conditions, pregnant females were strongly impacted by nightly temperature, returning earlier on colder nights (this effect was confirmed to be significant for pregnant females and not only significantly different from non‐reproductive females) (Table 1b; Figure 1e,f). These effects were all confirmed to be within‐subjects effects of individual plasticity (Table S2).

### 3.3 Proportion of Night Utilised

**FIGURE 2:**
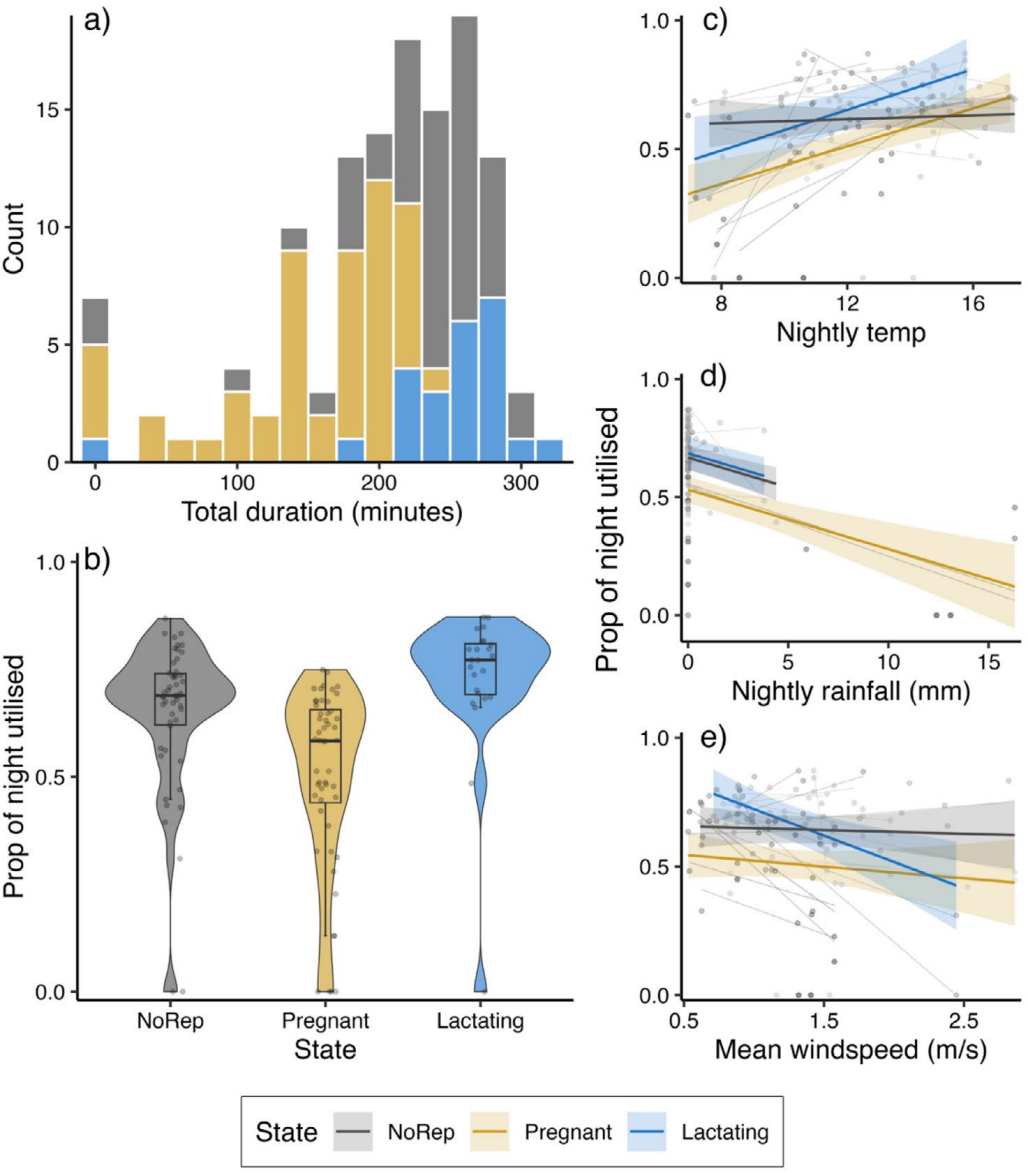
Variation in individual proportion of night utilised by female *Plecotus auritus* . (a) Histogram of the observations of total nightly durations from emergence to return across reproductive states. (b) Violin‐plots showing the distribution of proportion of the nighttime used. Medians, and 25th and 27th percentiles are shown within the violin plots. (c) Mean predicted effects and CIs of variation in proportion of night utilised explained by nightly mean temperature and individual reproductive state. Grey lines and datapoints indicate individual responses (raw‐data not accounting for other effects from the model). (d) Mean predicted effects and CIs of variation in proportion of night utilised explained by total nightly rainfall and individual reproductive state. (e) Predicted effects and CIs of variation in proportion of night utilised explained by mean nightly windspeed and individual reproductive state.

We recorded 126 individual nights (including seven observations where tagged bats did not fly out at night, that is, duration = 0 min, which is why the sample size is larger than the number of returns recorded) across 74 dates and 26 females. The nightly durations from emergence until return (excluding 0 duration observations) ranged from 40 min to 5.3 h (mean duration: 216 min ± 58.5 SD; Figure 2a), while the proportion of the night utilised ranged from 0.13 to 0.87 (mean proportion: 0.64 ± 0.16). On active nights, non‐reproductive females utilised on average 67% of the night (±13.0 SD; *N*_obs_ = 48), pregnant females utilised 54% (±15.7 SD; *N*_obs_ = 48) and lactating females utilised 75.7% of the night (±8.7 SD; *N*_obs_ = 23). The best final model (after model selection) for explaining variation in the proportion of the night utilised included a strong negative effect of total nightly rainfall (bats exploited less of the night on rainy nights), an interaction effect between reproductive state and mean nightly temperature conditions and an interaction effect between reproductive state and mean windspeed. Non‐reproductive females were not significantly affected by nightly temperature nor windspeed, while pregnant and lactating females were positively affected by temperature (using a larger proportion of the night on warmer nights; this effect was confirmed to be significant for pregnant and lactating females and not only significantly different from non‐reproductive females) and lactating females were also negatively affected by windspeed (Table 1c; Figure 2b–d). These effects were confirmed to be within‐subjects effects of individual plasticity (Table S3).

## 4 Discussion

Our results on the summer activity patterns in female *P. auritus* demonstrate strong responses to natural light conditions at roost exits, with delayed emergences on brighter evenings until light levels decreased below ~5 lux. However, we did not detect similar responses on the timing of returns to the roost, although *P. auritus* always returned before sunrise (except on one occasion) and generally before light levels exceeded 600 lux. As expected, we observed lactating females leaving the roost earlier than pregnant and non‐reproductive females in order to extend their foraging time. Lactating females also generally spent a larger proportion of the night away from the roost. Contrary to our predictions, lactating females were more sensitive to variation in temperature and wind‐conditions, as compared with non‐reproductive bats, while all bats were equally negatively impacted by increasing nightly rainfall regardless of reproductive state. The detailed patterns of individual plasticity in foraging behaviour observed in this population are consistent with a finely‐tuned behavioural strategy (see Fjelldal et al. 2023) that adaptively buffers individuals and populations from the consequences of living in a variable and harsh environment during reproductive periods with high energetic demands.

Bats in our study had an overall mean emergence time of 48 min after sunset, which is later than sympatric bat species at northern latitudes (Jones and Rydell 1994; Entwistle et al. 1996). A study on reproductive *E. nilssonii* from within the same vicinity as our study location in Norway found that the overall mean emergence time across the breeding season was 32 min after sunset (Lilley et al. 2025). The delay in emergence from the roost on brighter evenings could suggest considerable predation risks associated with leaving roosts during higher light‐levels, although a similar pattern was not observed between light‐levels and timing of returning to the roost. These results align with observations of attacks from diurnal avian raptors on bats mainly occurring around dusk or early evening (Frafjord 2012; Lima and O'Keefe 2013), explaining the pronounced light‐aversion in a slow‐flying gleaning species such as *P. auritus* (Entwistle et al. 1996).

This high‐latitude population appears to closely track the trade‐off between benefitting from a prolonged period of higher food abundance before night sets in by emerging earlier, versus the risk of being predated upon when leaving the roost (Speakman 1991b; Rydell 1993). On warmer evenings bats emerged earlier, thus risking earlier foraging flights to profit from the expected higher insect abundance following the daily temperature cycle, which is in accordance with observations from previous studies (Suutari et al. 2024; Lilley et al. 2025). Our findings of lactating females emerging earlier than non‐reproductive and pregnant females when accounting for environmental effects confirms our prediction of increasing energetic demands prompting individual bats into taking higher risks, as observed in other insectivorous bat species at high latitudes (Rydell 1993; Duvergé et al. 2000; Lilley et al. 2025).

Although all bats reduced their foraging durations on rainy nights, only reproductive females expressed sensitivity to nightly temperatures by extending their time away from the roost on warmer nights. This is contrary to our hypothesis of lactating females being less impacted by environmental conditions given their need to sustain a high energy intake. It could indicate that lactating females in this population track foraging profitability more closely than non‐reproductive bats, because of their higher energy needs and the strict requirement to keep a positive energy budget on a day‐to‐day basis whilst limiting any torpor use (Kurta et al. 1989). Pregnant females were also sensitive to nightly temperatures, but generally spent less time of the night away from the roost compared to lactating females. Reduced foraging activity during pregnancy has previously been observed in several bat species, likely due to heavier body mass increasing predation risk and flight costs (Speakman 1991b; McLean and Speakman 2000; Lilley et al. 2025). Pregnant females were observed to reduce their foraging duration mainly through earlier returns to the roost at night (Lilley et al. 2025; this study), although they also delayed their emergence time (Duvergé et al. 2000; this study), all of which was likely driven by trade‐offs against predation risk (Speakman 1991b; Jones and Rydell 1994). Our findings suggest that bats living in challenging northern environments balance their energy budgets not only through immediate responses to current conditions, but also through strategic evaluations given their state and their environment (see Fjelldal et al. 2023).

Strategic energy management in insectivorous bats has been observed in response to both individual energetic state (e.g., Wojciechowski et al. 2007; Matheson et al. 2010; Fjelldal et al. 2021, 2023; Sørås et al. 2022) and reproductive state (Duvergé et al. 2000; Dzal and Brigham 2013; Lilley et al. 2025). For example, the transition from gestation to lactation has been found to impose immediate and substantial shifts in activity patterns in bats while also being subject to more gradual temporal changes throughout the different reproductive phases (Duvergé et al. 2000; Lilley et al. 2025). However, we were only able to generalize the effects of individual reproductive state as we could not monitor the timing of parturition for each individual, and more fine‐scale temporal changes to activity patterns might therefore have been overlooked. Furthermore, although variation in individual energetic state is expected to influence summer foraging behavior in high‐latitude bats (Fjelldal et al. 2023), we could not test the effects of energetic reserves in our data given that we could not continuously measure body mass or foraging success in these free‐ranging individuals. Further studies investigating activity patterns in the field should perhaps attempt to collect body mass data at roosts and thus include more detailed individual states as potential predictors, as well as possibly test for the influence of risk of predation on emergence and activity (Suutari et al. 2024). Additionally, future studies could improve on this knowledge by collecting light measurements at individual roosts to disentangle the effects of light exposure between roost locations.

Our results further add to our understanding of how bats at high latitudes cope with restricted summer foraging times and how natural light conditions may be a limiting factor in future expansions of the distributional limits of bat species northward in response to climate change. Although the expected warmer temperature conditions in the sub‐arctic region could make thermal conditions more favorable, range shifts further north are therefore unlikely to be an option for many bats. Previous studies have proposed photoperiod as a limiting factor for high latitude bat species distributions in summertime (Parker et al. 1997; Slough and Jung 2008; Fjelldal et al. 2023), although the light‐tolerant species *E. nilssonii* has defied these expectations (Frafjord 2021). However, for light‐averse species, such as slow‐flying gleaning bats, our study demonstrates how individuals respond by delaying emergences until daylight levels are sufficiently reduced. The latitudes at which summers become energetically too demanding to survive due to shortened foraging nighttimes for such species are therefore likely reached before thermal summer environments limit distribution ranges in the north. This could potentially result in an overall range contraction for light‐sensitive species if climate conditions become unfavorable in the southern parts of their range.

## Author Contributions

**Mari A. Fjelldal:** conceptualization (equal), data curation (lead), formal analysis (lead), funding acquisition (supporting), investigation (equal), methodology (equal), visualization (lead), writing – original draft (equal). **Jonathan Wright:** conceptualization (equal), funding acquisition (lead), investigation (equal), methodology (supporting), project administration (equal), supervision (equal), validation (equal), writing – review and editing (equal). **Thomas M. Lilley:** conceptualization (supporting), investigation (equal), methodology (equal), validation (equal), writing – original draft (equal). **Rune Sørås:** data curation (supporting), investigation (equal), methodology (supporting), validation (equal), writing – review and editing (equal). **Clare Stawski:** conceptualization (equal), data curation (supporting), funding acquisition (lead), investigation (equal), methodology (supporting), project administration (equal), supervision (equal), validation (equal), writing – review and editing (equal).

## Conflicts of Interest

The authors declare no conflicts of interest.

## Supporting information


Data S1.

## Data Availability

Data and r‐codes have been uploaded to the online data repository Zenodo. DOI: https://doi.org/10.5281/zenodo.15622724.
