# Supplementary material for: Female Brown Long‐Eared Bats (Plecotus auritus) Delay Roost Emergence at Elevated Natural Light Conditions
Source: Ecol Evol. 2025 Jun 29;15(7):e71699. doi: 10.1002/ece3.71699 (PMC12206950; doi:10.1002/ece3.71699)
Supplement: Supplementary file 1 — Data S1. [file ECE3-15-e71699-s001.docx]

**Supplementary Materials**

Distinguishing within- versus between-subject effects

To separate within-subject effects from between-subject effects, we applied the within-subject centering method described in van de Pol and Wright (2009) on the final best-fit models (after model selection). Model results presenting original (step 1) and decomposed within- and among-subject effects (step 2 and 3) explaining variation in emergence time relative to sunset, return time relative to sunrise, and total nightly foraging duration are shown in Table S1, S2 and S3, respectively.

**Table S1:** Model results presenting original (step 1) and decomposed within- and among-subject effects (step 2 and 3; see van de Pol and Wright (2009)) from the final best-fit model (after model selection) explaining variation in the timing of emergence relative to sunset. Intercepts and their *p* values are given in relation to the first listed intercept value, while interaction effects and their *p* values for pregnant and lactating females signifies whether the effects are different from the effect for non-reproductive females. Bold values indicate significant effects (p value < 0.05). The indication of (w) indicates within-subject effects, while (a) indicates among-subject effects. The notion (a-w) indicates the difference between the among- and within-subjects effect.

| **Model: timing of emergence (minutes since sunset)** | | | | | | |
| --- | --- | --- | --- | --- | --- | --- |
|  | Step 1 (original) | | Step 2 (w. & a.) | | Step 3 (w. vs. a.) | |
| *Random effects* | | | | | | |
| Variable | Variance (sd) | | Variance (sd) | | Variance (sd) | |
| Individual ID | 39.8 (6.3) | | 38.5 (6.2) | | 38.5 (6.2) | |
| Date | 0.0 (0.0) | | 0.0 (0.0) | | 0.0 (0.0) | |
| Roost ID | 293.5 (17.1) | | 281.7 (16.8) | | 281.7 (16.8) | |
| Residual | 252.8 (15.9) | | 252.1 (15.9) | | 252.1 (15.9) | |
| *Fixed effects* | | | | | | |
| Variable | Estimate (se) | *p* value | Estimate (se) | *p* value | Estimate (se) | *p* value |
| Intercept (non-rep) | -18.0 (46.3) | 0.6983 | 14.4 (68.3) | 0.8339 | 14.4 (68.3) | 0.8339 |
| Intercept (pregnant) | **15.8 (7.1)** | 0.0324 | 14.1 (7.5) | 0.0690 | 14.1 (7.5) | 0.0690 |
| Intercept (lactating) | -8.0 (7.3) | 0.2766 | -9.8 (7.4) | 0.1961 | -9.8 (7.4) | 0.1961 |
| T_a_ at sunset | **-2.9 (0.6)** | < 0.001 |  |  |  |  |
| T_a_ at sunset (w) |  |  | **-3.1 (0.8)** | < 0.001 | **-3.1 (0.8)** | < 0.001 |
| T_a_ at sunset (a) |  |  | -1.9 (1.3) | 0.1525 |  |  |
| T_a_ at sunset (a-w) |  |  |  |  | 1.1 (1.5) | 0.4626 |
| Lux at sunset | **1.3 (0.3)** | < 0.001 |  |  |  |  |
| Lux at sunset (w) |  |  | **1.4 (0.4)** | < 0.001 | **1.4 (0.4)** | < 0.001 |
| Lux at sunset (a) |  |  | -0.6 (0.9) | 0.5116 |  |  |
| Lux at sunset (a-w) |  |  |  |  | -0.8 (1.0) | 0.3843 |
| Night length | **16.4 (7.6)** | 0.0337 |  |  |  |  |
| Night length (w) |  |  | 19.1 (13.5) | 0.1600 | 19.1 (13.5) | 0.1600 |
| Night length (a) |  |  | 9.7 (11.1) | 0.3864 |  |  |
| Night length (a-w) |  |  |  |  | -9.4 (16.0) | 0.5568 |

**Table S2:** Model results presenting original (step 1) and decomposed within- and among-subject effects (step 2 and 3) from the final best-fit model explaining variation timing of return relative to sunrise. Bold values indicate significant effects (p value < 0.05). Intercepts and their *p* values are given in relation to the first listed intercept value, while interaction effects and their *p* values for pregnant and lactating females signifies whether the effects are different from the effect for non-reproductive females. The indication of (w) indicates within-subject effects, while (a) indicates among-subject effects. The notion (a-w) indicates the difference between the among- and within-subjects effect.

| **Model: timing of returns (minutes since sunset)** | | | | | | |
| --- | --- | --- | --- | --- | --- | --- |
|  | Step 1 (original) | | Step 2 (w. & a.) | | Step 3 (w. vs. a.) | |
| *Random effects* | | | | | | |
| Variable | Variance (sd) | | Variance (sd) | | Variance (sd) | |
| Individual ID | 110.7 (10.5) | | 74.5 (8.6) | | 74.5 (8.6) | |
| Date | 335.9 (18.3) | | 315.5 (17.8) | | 315.5 (17.8) | |
| Roost ID | 38.2 (6.2) | | 130.5 (11.4) | | 130.5 (11.4) | |
| Residual | 873.5 (29.6) | | 815.3 (28.6) | | 815.3 (28.6) | |
| *Fixed effects* | | | | | | |
| Variable | Estimate (se) | *p* value | Estimate (se) | *p* value | Estimate (se) | *p* value |
| Intercept (non-rep) | **96.4 (28.5)** | 0.0012 | **134.2 (39.0)** | 0.0011 | **134.2 (39.0)** | 0.0011 |
| Intercept (pregnant) | **92.0 (39.2)** | 0.0215 | 69.7 (56.2) | 0.2276 | 69.7 (56.2) | 0.2276 |
| Intercept (lactating) | -43.2 (56.6) | 0.4475 | **-178.9 (74.4)** | 0.0213 | **-178.9 (74.4)** | 0.0213 |
| Total rainfall | **6.3 (1.6)** | < 0.001 |  |  |  |  |
| Total rainfall (w) |  |  | **6.5 (1.6)** | < 0.001 | **6.5 (1.6)** | < 0.001 |
| Total rainfall (a) |  |  | -2.6 (6.2) | 0.6798 |  |  |
| Total rainfall (a-w) |  |  |  |  | -9.1 (6.4) | 0.1662 |
| Mean T_a_ | -2.4 (2.2) | 0.2683 |  |  |  |  |
| Mean T_a_ (w) |  |  | -0.3 (2.7) | 0.9060 | -0.3 (2.7) | 0.9060 |
| Mean T_a_ (a) |  |  | -5.3 (3.0) | 0.0964 |  |  |
| Mean T_a_ (a-w) |  |  |  |  | -4.9 (3.9) | 0.2086 |
| T_a_ × pregnant | **-6.7 (3.0)** | 0.0281 |  |  |  |  |
| T_a_ × pregnant (w) |  |  | **-8.6 (3.6)** | 0.01931 | **-8.6 (3.6)** | 0.01931 |
| T_a_ × pregnant (a) |  |  | -5.0 (4.3) | 0.2594 |  |  |
| T_a_ × pregnant (a-w) |  |  |  |  | 3.6 (5.4) | 0.5065 |
| T_a_ × lactating | 2.4 (4.4) | 0.5835 |  |  |  |  |
| T_a_ × lactating (w) |  |  | -6.7 (6.1) | 0.2789 | -6.7 (6.1) | 0.2789 |
| T_a_ × lactating (a) |  |  | **13.5 (5.9)** | 0.0284 |  |  |
| T_a_ × lactating (a-w) |  |  |  |  | **20.2 (8.2)** | 0.0163 |

**Table S3:** Model results presenting original (step 1) and decomposed within- and among-subject effects (step 2 and 3) from the final best-fit model explaining variation in proportion of the night utilised. Bold values indicate significant effects (p value < 0.05). Intercepts and their *p* values are given in relation to the first listed intercept value, while interaction effects and their *p* values for pregnant and lactating females signifies whether the effects are different from the effect for non-reproductive individuals. The indication of (w) indicates within-subject effects, while (a) indicates among-subject effects. The notion (a-w) indicates the difference between the among- and within-subjects effect.

| **Model: proportion of the night utilised** | | | | | | |
| --- | --- | --- | --- | --- | --- | --- |
|  | Step 1 (original) | | Step 2 (w. & a.) | | Step 3 (w. vs. a.) | |
| *Random effects* | | | | | | |
| Variable | Variance (sd) | | Variance (sd) | | Variance (sd) | |
| Individual ID | 0.002 (0.04) | | 0.002 (0.04) | | 0.002 (0.04) | |
| Date | 0.01 (0.12) | | 0.01 (0.12) | | 0.01 (0.12) | |
| Roost ID | 0.0 (0.0) | | 0.0 (0.0) | | 0.0 (0.0) | |
| Residual | 0.006 (0.08) | | 0.005 (0.07) | | 0.005 (0.07) | |
| *Fixed effects* | | | | | | |
| Variable | Estimate (se) | *p* value | Estimate (se) | *p* value | Estimate (se) | *p* value |
| Intercept (non-rep) | **0.64 (0.15)** | < 0.001 | **0.64 (0.23)** | 0.0091 | **0.64 (0.23)** | 0.0091 |
| Intercept (pregnant) | **-0.51 (0.19)** | 0.0077 | -0.54 (0.30) | 0.0859 | -0.54 (0.30) | 0.0859 |
| Intercept (lactating) | -0.09 (0.20) | 0.6522 | 0.26 (0.30) | 0.3943 | 0.26 (0.30) | 0.3943 |
| Total rainfall | **-0.03 (0.006)** | < 0.001 |  |  |  |  |
| Total rainfall (w) |  |  | **-0.02 (0.006)** | < 0.001 | **-0.02 (0.006)** | < 0.001 |
| Total rainfall (a) |  |  | -0.02 (0.01) | 0.1639 |  |  |
| Total rainfall (a-w) |  |  |  |  | 0.01 (0.02) | 0.5240 |
| Mean T_a_ | 0.004 (0.009) | 0.6592 |  |  |  |  |
| Mean T_a_ (w) |  |  | -0.007 (0.01) | 0.5484 | -0.007 (0.01) | 0.5484 |
| Mean T_a_ (a) |  |  | 0.02 (0.01) | 0.1579 |  |  |
| Mean T_a_ (a-w) |  |  |  |  | -0.002 (0.01) | 0.9036 |
| Mean wind | -0.01 (0.04) | 0.7242 |  |  |  |  |
| Mean wind (w) |  |  | -0.009 (0.04) | 0.8379 | -0.009 (0.04) | 0.8379 |
| Mean wind (a) |  |  | -0.14 (0.11) | 0.2287 |  |  |
| Mean wind (a-w) |  |  |  |  | 0.01 (0.06) | 0.8658 |
| T_a_ × pregnant | **0.03 (0.01)** | 0.0049 |  |  |  |  |
| T_a_ × pregnant (w) |  |  | **0.05 (0.01)** | < 0.001 | **0.05 (0.01)** | < 0.001 |
| T_a_ × pregnant (a) |  |  | 0.02 (0.02) | 0.2787 |  |  |
| T_a_ × pregnant (a-w) |  |  |  |  | -0.006 (0.02) | 0.7607 |
| T_a_ × lactating | **0.03 (0.01)** | 0.0439 |  |  |  |  |
| T_a_ × lactating (w) |  |  | **0.06 (0.02)** | 0.0023 | **0.06 (0.02)** | 0.0023 |
| T_a_ × lactating (a) |  |  | 0.003 (0.02) | 0.8828 |  |  |
| T_a_ × lactating (a-w) |  |  |  |  | -0.03 (0.03) | 0.3459 |
| Wind × pregnant | -0.03 (0.06) | 0.6026 |  |  |  |  |
| Wind × pregnant (w) |  |  | -0.06 (0.07) | 0.3990 | -0.06 (0.07) | 0.3990 |
| Wind × pregnant (a) |  |  | 0.14 (0.13) | 0.2991 |  |  |
| Wind × pregnant (a-w) |  |  |  |  | 0.01 (0.11) | 0.9135 |
| Wind × lactating | **-0.19 (0.06)** | 0.0035 |  |  |  |  |
| Wind × lactating (w) |  |  | **-0.15 (0.06)** | 0.0212 | **-0.15 (0.06)** | 0.0212 |
| Wind × lactating (a) |  |  | -0.24 (0.17) | 0.1598 |  |  |
| Wind × lactating (a-w) |  |  |  |  | -0.09 (0.18) | 0.6166 |

van de Pol, M. and Wright, J. 2009. A simple method for distinguishing within-versus between-subject effects using mixed models. - Anim. Behav. 77: 753-758.
